# Large scale structure-function mappings of the human subcortex

**DOI:** 10.1038/s41598-018-33796-y

**Published:** 2018-10-26

**Authors:** Max C. Keuken, Leendert van Maanen, Michiel Boswijk, Birte U. Forstmann, Mark Steyvers

**Affiliations:** 1 University of Amsterdam, Integrative Model-based Cognitive Neuroscience research unit, Amsterdam, The Netherlands; 2 University of Leiden, Cognitive Psychology, Leiden, The Netherlands; 3 University of Amsterdam, Department of Psychological Methods, Amsterdam, The Netherlands; 4 Department of Cognitive Sciences, University of California, Irvine, USA

## Abstract

Currently little is known about structure-function mappings in the human subcortex. Here we present a large-scale automated meta-analysis on the literature to understand the structure-function mapping in the human subcortex. The results provide converging evidence into unique large scale structure-function mappings of the human subcortex based on their functional and anatomical similarity.

## Introduction

Approximately a quarter of the human brain consists of the subcortex^1^ but is to a large extent still uncharted territory^2^. An important reason for this knowledge gap is that the human subcortex is notoriously difficult to visualize and analyze with functional magnetic resonance imaging (fMRI) because subcortical nodes are relatively small and lie relatively far away from the receiver coils of the MRI scanner^3,4^. Moreover, only 7% of subcortical structures are depicted in standard MRI-atlases^2,3,5^, making the subcortex challenging to study using standard MRI pipelines.

To gain an understanding of the mapping of cognitive functions to subcortical structures, we hypothesized that structure can inform function^3,6–8^. Testing this prediction with meta-analytical tools such as ALE or Neurosynth^9,10^ is, however, challenging for the subcortex and methods are based on smoothed activation maps in a standard brain.

To understand the structure-function mapping in the subcortex, we applied a novel approach based on an automated analysis of structure and function terms found in the literature. To this end, we harvested publications from the PubMed database that were associated with subcortical grey matter structures, with a functional neuroimaging technique (e.g., fMRI), and that studied humans. We conducted a large-scale automated meta-analysis on the literature to (1) find the subcortical structures that are studied the most, (2) find what - if any - are the cognitive functions most commonly associated with the human subcortex, (3) understand if there is a similarity between the cognitive functions implemented in subcortical regions, and (4) whether this functional similarity can be explained by the anatomical similarity between subcortical regions. These publications were classified according to their assigned Medical Subject Headers (MeSH topics). The MeSH ontology contains hierarchically ordered topics, that include cognitive and non-cognitive topics. We validated our results with a probabilistic topic model^8,11,12^, constructed from the keywords present in the title and abstracts of the publications, and therefore independent of the MeSH ontology.

## Results

**Figure 1:**
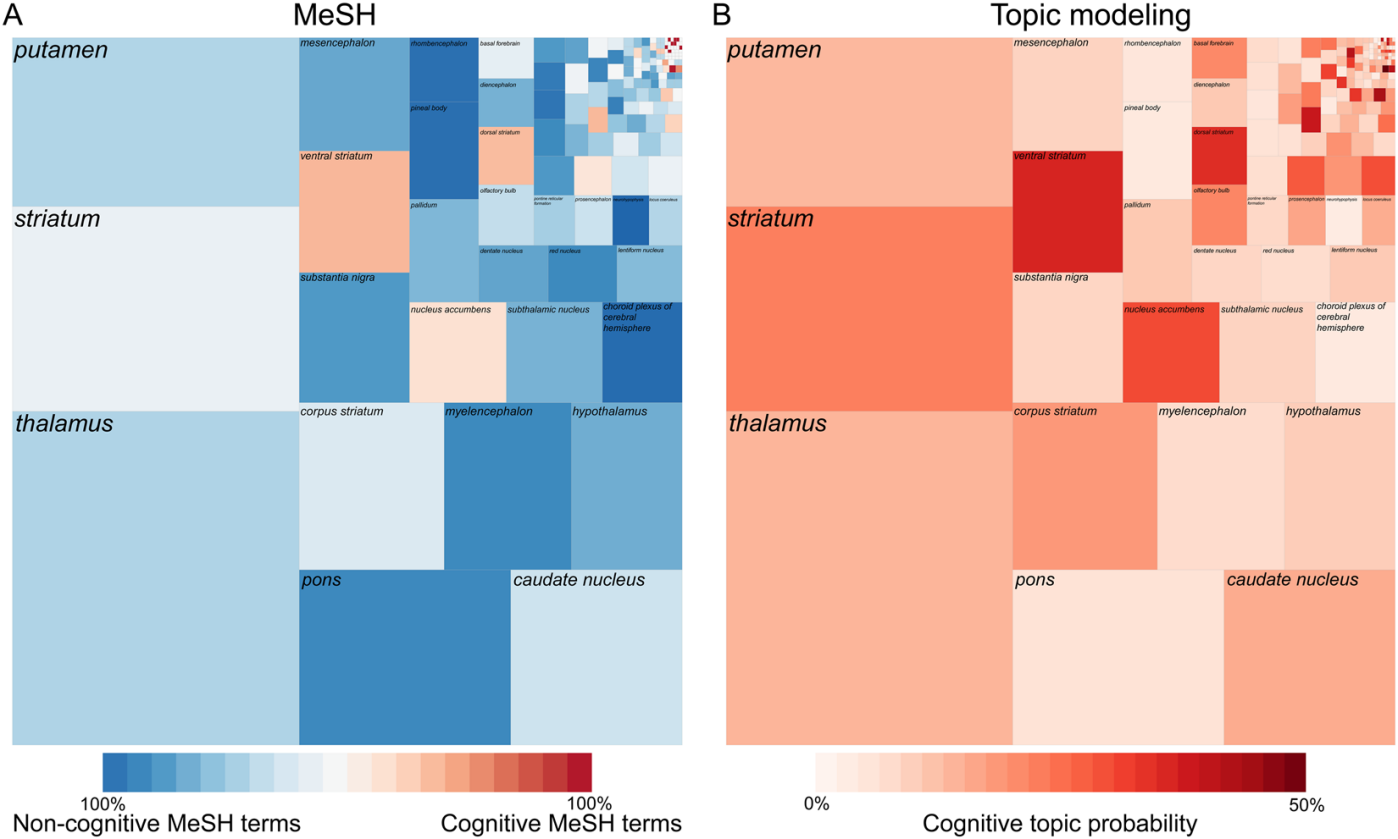
A few structures account for most of the retrieved publications, but usage of cognitive versus non-cognitive topics do not depend on the ontology. The area associated with each of those 145 structures is based on the number of unique publications found with this query. (**A**) The color coding reflects the proportion of publications that contained MesH topics associated with cognitive terms for each structure. (**B**) The color coding reflects the proportion of publications that contained data-driven topics associated with cognitive terms for each structure.

The PubMed search query resulted in approximately 37k unique publications for 145 of the 424 known subcortical structures, of which 103 were associated with a *Psychological phenomena and processes* MeSH topic. Fig. 1A shows that a few structures (thalamus, striatum, putamen, pons, caudate nucleus) account for most of the publications (57% of the corpus). These structures, as well as many others, are predominantly associated with non-cognitive MeSH topics. There are, however, some structures (e.g., the ventral striatum, nucleus accumbens, and dorsal striatum) which are associated with more cognitive MeSH topics than with non-cognitive topics. The non-cognitive versus cognitive loading per structure did not heavily depend on the ontology that was used. This MeSH topic result was replicated using probabilistic topic modeling^12^ that resulted in a very similar square tree map (Fig. 1B). The Spearman rank-correlation between the proportion of cognitive topics as defined by the MeSH ontology and the proportion of cognitive topics based on the probabilistic topic modeling for all 145 subcortical structures that had more than one publications was ρ = 0.50 (S = 251920, p < 0.001).

**Figure 2:**
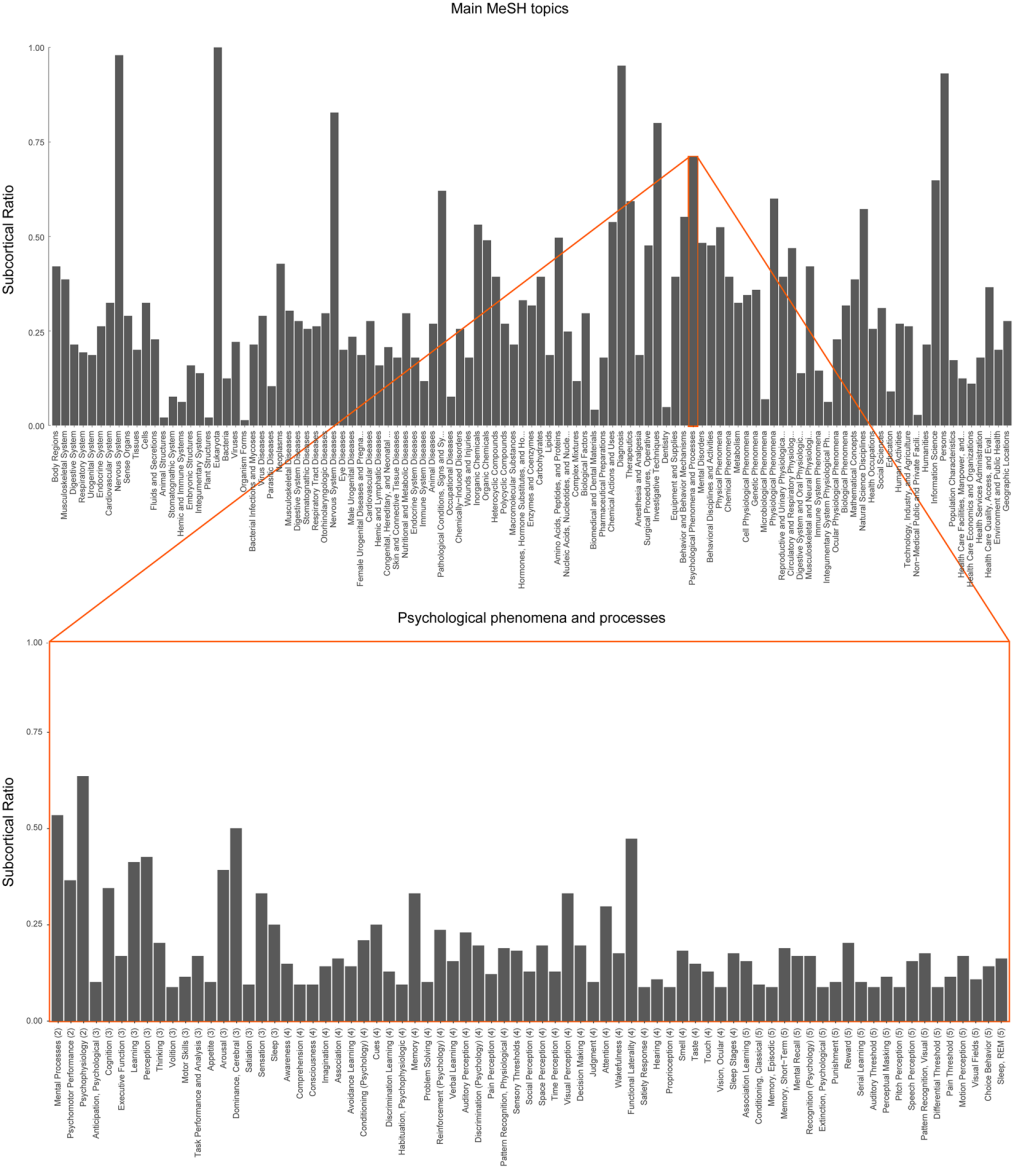
Proportion of subcortical structures associated with the MeSH topics. The proportion of the 145 subcortical structures that are associated with a given MeSH topic (x-axis). Top panel: The ratio for the top-level MeSH topics are displayed. Bottom panel: The ratio for the most frequently associated cognitive subtopics of the “*Psychological phenomena and processes*” branch are displayed. For visualization purposes only, we selected the “*Psychological phenomena and processes*” topics that had at least 13 publications associated with them. Note that this number was arbitrarily chosen to ensure manageable figure dimensions. The data and code to generate the figure without this filter can be found on osf.io/nf482.

There seems to be substantial variability in the proportion of subcortical structures associated with a given MeSH topic. As is shown in the top panel of Fig. 2, only the top-level MeSH topic *Eukaryota* is associated with all 145 structures for which we have data. Top-level MeSH topics such as *Nervous System, Nervous System Diseases*, and *Psychological phenomena and processes* are associated with most of the 145 subcortical structures, whereas topics as *Dentistry* and *plant structures* generally seem to have lower associations. Similarly, within the *Psychological phenomena and processes* MeSH topics there are a number of topics such as *Mental Processes, Psychomotor Performance*, and *Arousal* that are associated with a substantial portion of the subcortical areas, whereas topics such as *Volition* and *Appetite* are more sparsely associated (Fig. 2, bottom panel).

**Figure 3:**
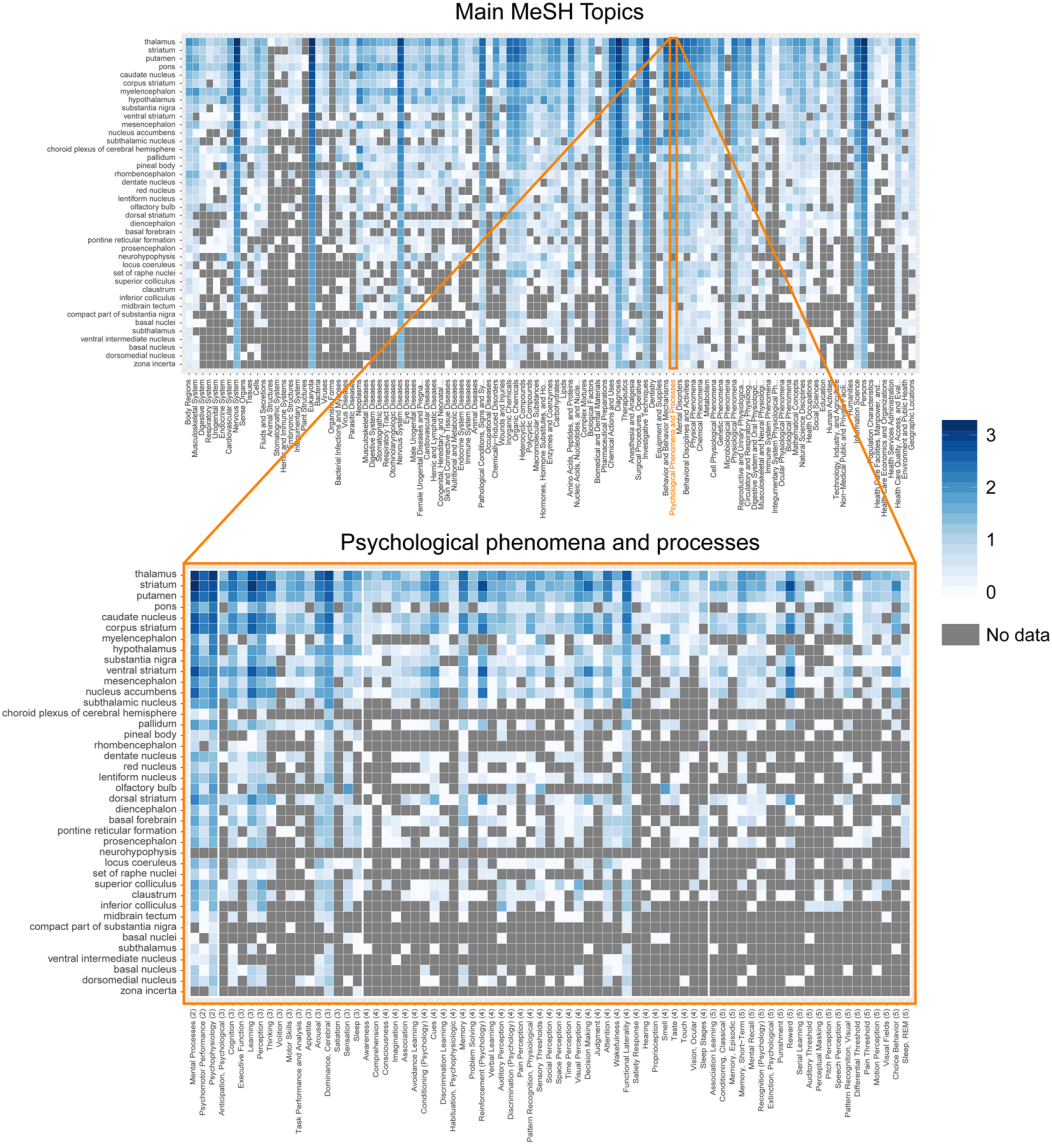
Structure-function mapping of the human subcortex. MeSH topics (x-axis) are shown for the subcortical structures with the highest number of unique publications (y-axis). Color shading indicates the number of publications for each structure-function pair. Top panel: The top main MeSH topics are displayed including the main *Psychological phenomena and processes* topic. Bottom panel: The top cognitive subtopics of the *Psychological phenomena and processes* branch. For visualization purposes only, we selected the *Psychological phenomena and processes* topics that had at least 13 publications associated with them. Note that this number was arbitrarily chosen to ensure manageable figure dimensions. The data and code to generate the figure without this filter can be found on osf.io/nf482. Values are cumulative according to the hierarchical structure of the MeSH ontology (different levels of the ontology are indicated by wider vertical spacing). Note that a similar figure is not possible for the topic model as the hierarchical topic structure is lacking.

For the 40 anatomical structures with the highest number of publications (98% of the corpus) we plotted the number of MeSH topics per top-level topic of the MeSH tree (Fig. 3, top panel) and separately for the *Psychological phenomena and processes* subtopic, which is used to define cognitive topics (Fig. 3, bottom panel). It is clear that the well-studied structures (e.g., the thalamus) are related to a large number of non-cognitive topics but also to a wide range of cognitive topics. Also, some level of specification can be observed: For example, relative to the other cognitive phenomena, the dorsal striatum seems to be mostly associated with learning, reinforcement, and reward, whereas the mesencephalon is dominated by sensation-related topics. Interestingly, the ventral striatum seems to have a similar functional profile as the dorsal striatum which is in line with the fact that sharp anatomical borders seem to be absent between the two regions^13^.

**Figure 4:**
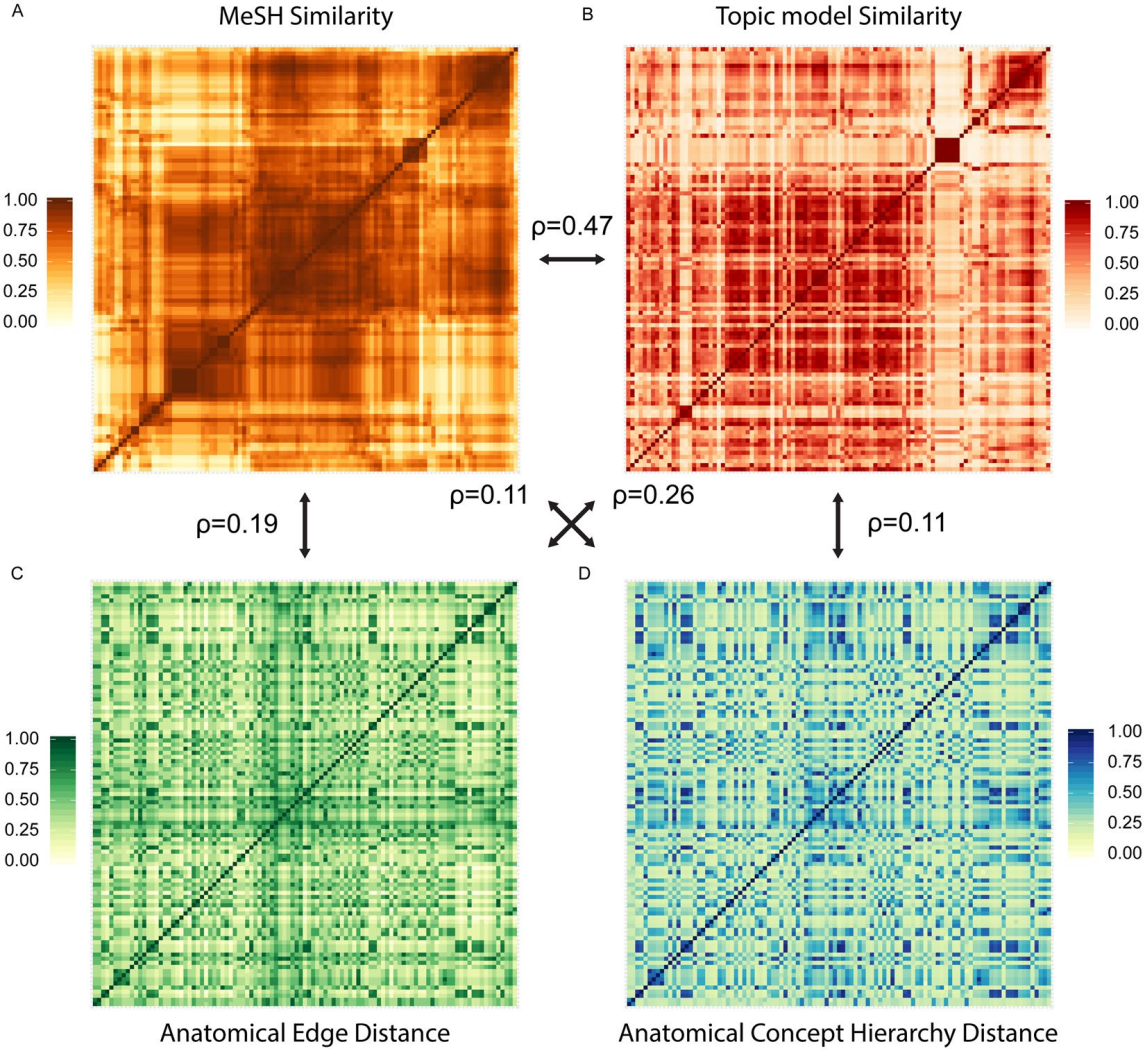
The similarity matrices for the 103 structures that had at least one psychological phenomenon or process associated with it. (**A**) The MeSH functional similarity matrix. (**B**) The Topic modeling functional similarity matrix. (**C**) The anatomical similarity matrix as defined by the edge distance. (**D**) The anatomical similarity matrix as defined by the concept hierarchy distance. The MeSH similarity matrix (panel A) is organized using a hierarchical clustering algorithm to optimize the visualization of the internal block structure. The other three matrices in panel (B–D) have been reorganized to have the identical organization as the MeSH similarity matrix. The arrows and corresponding ρ values indicate the correlation between the different matrices.

We then tested whether there is a relationship between the functional similarities of subcortical structures as defined by the co-occurrence of MeSH topics. As shown in Fig. 4A, there is a clear organization of the functional MeSH similarity matrix, indicating that a number of subcortical areas are more similar in function to each other than to others. A similar functional similarity between subcortical structures was found when using the topic model (Fig. 4B), which correlated with the MeSH topic similarity (ρ = 0.47, S = 2.145^e+10^, p < 0.001). The functional similarity matrix was generated by first creating a vector indicating the load of a given functional label per anatomical structure. This was done both for the *Psychological phenomena and processes* MeSH topics and the psychological topic probability models. The functional similarity matrices were generated by correlating the associated functional vector for a given brain structure with any of the other brain structures. A possible explanation why certain structures are functionally similar could be their common developmental origin, i.e., anatomical similarity^14^. Anatomical similarity is defined as the hierarchical edge distance in the anatomical terminology by the Federative Community on Anatomical Terminology (FCAT) (Fig. 4C). The hierarchical anatomical terminology provided by FCAT is organized according to embryonic vertebrate brain development and topographical rules^15^. In the method section we provide a visualization of a small section of the FCAT hierarchical tree and the corresponding anatomical similarity (see the *Anatomical similarity* section of the methods). This hypothesis was tested by correlating the anatomical similarity as defined by edge-counting with the functional similarity. We found a positive correlation between the MeSH similarity matrix (Fig. 4A) and the anatomical edge distance similarity matrix (Fig. 4C; ρ = 0.19, S = 1.9533^e+10^, p < 0.001). This suggest that if the MeSH topics of two structures are comparable, their anatomical hierarchical distance is also comparable. Using a functional similarity measure derived from the probabilistic topic model (Fig. 4B) again resulted in a similar positive correlation with the anatomical edge-counting distance (Fig. 4C; ρ = 0.26, S = 1.7974^e+10^, p < 0.001). Similarly, when determining the anatomical similarity by controlling for taxonomic hierarchical level (Fig. 4D) we find a positive, albeit lower, correlation with MeSH similarities (ρ = 0.11, S = 2.145^e+10^, p < 0.001) and topic model similarity (ρ = 0.11, S = 2.1607^e+10^, p < 0.001). See the method section *Anatomical similarity* for the difference between edge-counting and account for the taxonomic hierarchical levels.

**Figure 5:**
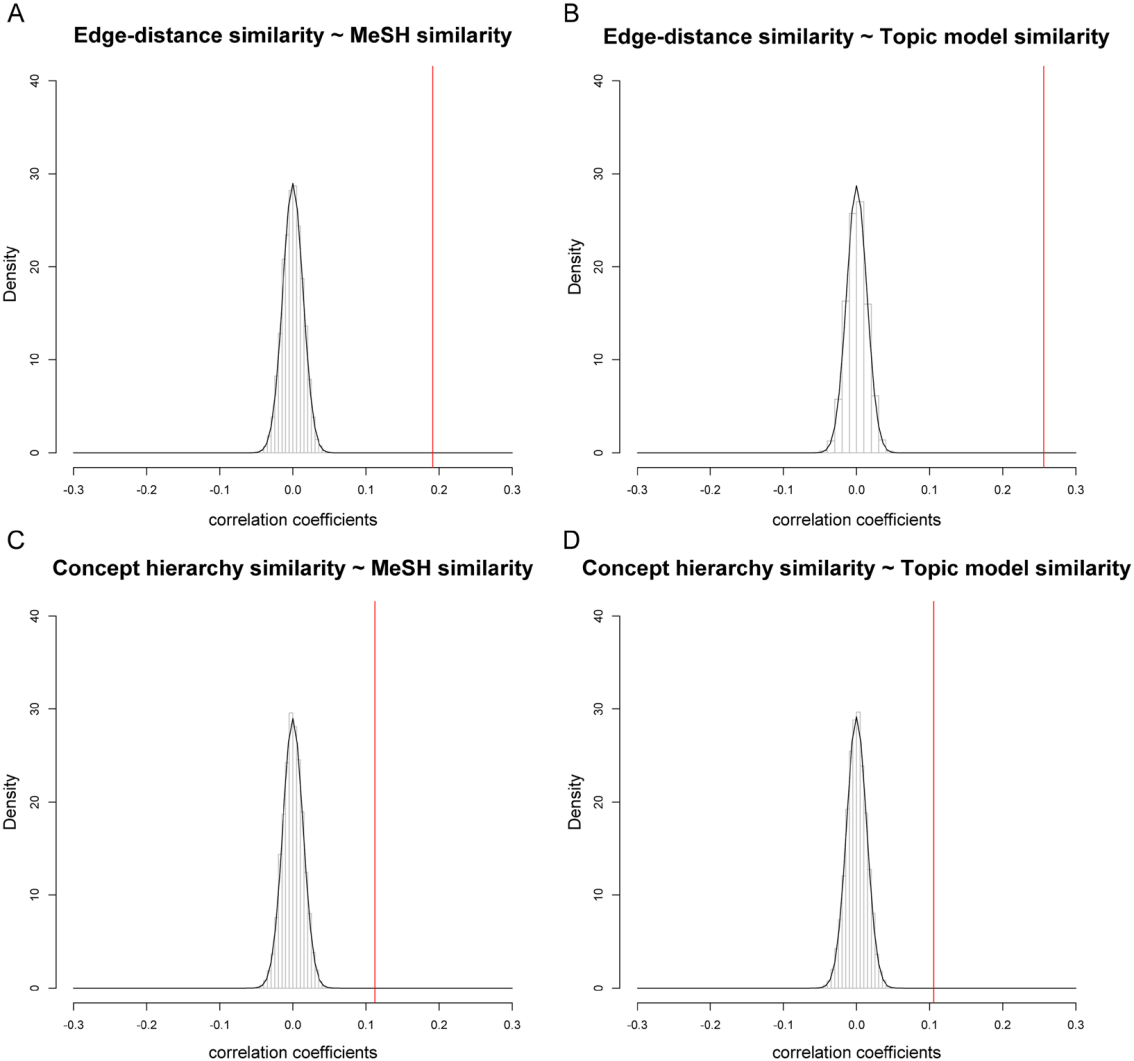
The correlation coefficient of anatomical and functional similarity relative to a null distribution. (**A**) The correlation between the anatomical similarity as defined by the edge-distance and MeSH similarity is 13.8SD removed from the null distribution. (**B**) The correlation between the anatomical similarity as defined by the edge-distance and Topic model similarity is 18.6SD removed from the null distribution. (**C**) The correlation between the anatomical similarity as defined by the concept hierarchy and MeSH similarity is 8.1SD removed from the null distribution. (**D**) The correlation between the anatomical similarity as defined by the edge-distance and probability topic model similarity is 7.6SD removed from the null distribution. The red line indicates the true correlation coefficient between the anatomical and functional matrices.

The final analysis was done to test how spurious the similarity correlations were by creating a correlation null distribution. The results indicate that the correlation between the anatomical similarity as defined by edge-counting and the MeSH similarity and topic model similarity is 13.8 SD and 18.6 SD removed from the permuted null distribution (Fig. 5A,B). The correlation between the anatomical similarity as defined by the concept hierarchy, and the MeSH and topic model similarity is 8.1 SD and 7.6 SD, respectively, removed from the permuted null distribution (Fig. 5C,D), making it unlikely that these results are due to chance. Therefore, while the correlation coefficients are low, the anatomical similarity between areas seems to be informative of the functional similarity.

## Discussion

We found that only a small proportion of subcortical structures is systematically studied using functional neuroimaging techniques, and that the majority of those studies do not relate to cognitive function terms, defined by either the MeSH ontology, or a data-driven topic model. The absence of the bulk of subcortical structures might be explained by the fact that only a small percentage of subcortical structures is accessible using standard MRI atlases^2,16^. A potential solution for this discrepancy is the use of individual anatomy to identify the subcortical structures per individual^3^. Such an approach is increasingly more feasible with the recent advancement of ultra-high field MRI (7 Tesla and higher), as the subcortical structures can be more easily distinguished from their direct surrounding^4,17,18^. A noteworthy observation is that it seems that subcortical brain structures that are easier to visualize with MRI are more often discussed in the literature. Although we cannot quantify this observation because of a lack of volume estimates of many of the structures, this could have potentially biased our results. Future work could focus on charting these smaller subcortical areas^3^ such that (1) volumetric estimates become available, and (2) probabilistic atlases are created. Together with increased spatial resolution in ultra high-field imaging^17,18^, this would allow for targeted functional imaging of small subcortical nuclei. It is our belief that such a combined approach will close the gap in our understanding of structure-function mapping of the human subcortex.

We also found that certain cognitive subcortical networks seem to exist, in that cognitive terms co-occur in subsets of the subcortical regions. As demonstrated in Fig. 3, there may, however, not be a simple one-to-one mapping of cognitive functions to subcortical structures. For instance, the dorsal striatum seems to be associated mostly, but not exclusively, with learning, reinforcement, and reward. This makes it challenging to infer cognitive functions from neuroimaging data, which is also known as the reverse inference problem^8,19^. One approach to address this problem is to account for the likelihood of activation during a task given the base rate activation across tasks^19–21^. Meta-analytic tools such as Neurosynth can provide reverse inference statistical maps^9^. Such maps can be used to estimate the relative selectivity with which a region activates, controlled for the base rate of activation across tasks. Our approach is complementary to such reverse inference maps as we can illustrate the functional-anatomical similarity across regions. Taking both the activation selectivity within and between regions into account, one can start to infer the structure-function relationship in the brain.

### Limitations

There are a few limitations of our study that need to be addressed. Firstly, the anatomical nomenclature of the FCAT is not universally adopted by the scientific community^22,23^. To illustrate this, the search query for the *ventral tegmental nucleus* only resulted in a single hit. This was surprising as this nucleus contains a large number of dopaminergic neurons which are thought to be crucial for a wide range of cognitive functions^24^. The lack of hits can be explained by the fact that the scientific community uses a slightly different term, namely the ventral tegmental *area*, which is not incorporated in the FCAT. Secondly, another limitation relates to the hierarchical structure as a proxy for anatomical similarity as it does not take the actual spatial proximity into account. Especially with the use of relatively large smoothing kernels in fMRI the chance of misattributing the BOLD signal - and therefore function to adjacent structures - becomes problematic^25^. Such a mixture of structure-function associations might hinder the one-to-one mapping of function to structure. A control analysis for such a spatial mixture would entail an anatomical similarity index based on the relative spatial location. Another control analysis would be to incorporate diffusion weighted imaging (DWI) and resting-state fMRI data to determine the connectivity between anatomical structures that have a high functional similarity. These two control analyses are, however, currently not feasible as only a fraction of the subcortical structures are mapped in standard anatomical MRI atlases^2,3^. A final limitation concerns the structures that were not associated with any *Psychological phenomena and processes* MeSH term. There is a certain (unknown) probability that these structures are in fact associated with psychological phenomena or processes, but are not in included in the analysis because there are no papers that study this association. Our similarity matrices do not account for this probability, and in particular do not account for the probability that this occurs in a specific non-random subset of structures. This may be due to the fact that the literature is biased towards larger structures. Whether the functional similarities that we identify and the structure-function mappings extend to these non-studied subcortical areas is a matter of future research.

## Conclusion

The results give insight into unique large scale structure-function mappings of the human subcortex and have the potential to generate functional predictions for lesser known structures based on their anatomical similarity.

## Materials and Methods

### Pubmed search query

A comprehensive literature search was conducted using the Entrez search tools implemented in the Biopython’s Bio.Entrez module^26^. This is a python application programming interface (API) tool that queries the PubMed database (www.pubmed.org). The query date was the 28^th^ of June 2017 and used the following search query: *Subcortical structure name* AND (MRI OR fMRI OR PET OR electrophysiology) AND (humans OR humans [MeSH Terms]). The search query was specified with a list of subcortical structure names from the Federative Community on Anatomical Terminology (FCAT)^5^. We queried English and Latin spelling of each structure and the corresponding officially acknowledged equivalent or synonyms. This list and the corresponding hierarchical order of these subcortical structures can be found online (www.unifr.ch/ifaa/Public/EntryPage/ShowTA98EN.html). The different spellings and synonyms were included to ensure a comprehensive search but did result in a number of duplicate publications. To not bias the results, all duplicates were removed from further analysis. The search query resulted in 37,367 unique publications for 145 of the 424 FCAT structures, of which 103 were associated with a *Psychological phenomena and processes* MeSH topic. The list of structures for which we did not find any publication include structures such as periventricular nucleus, the sub nuclei of the red nucleus, and the tail of the caudate nucleus. The entire list of 279 missing structures can be found on www.OSF.io/nf482. It was surprising not to find any publications for the periventricular nucleus as it is an area which is frequently targeted with deep brain stimulation to alleviate intractable pain^27^. The resulting unique PubMed ID, title, abstract, and corresponding Medical Subject Header (MeSH, see below) terms were combined per structure and used for further analysis.

### MeSH ontology

The MeSH ontology is a set of terms in a hierarchical structure that is manually curated by the U.S. National Library of Medicine and describes a broad range of terms that occur in the scientific medical literature. Each entry on PubMed is (or is in the process of being) associated with a set of MeSH terms from this ontology. The MeSH hierarchical tree can be found online (https://www.nlm.nih.gov/mesh/2017/download/2017MeshTree.txt) and was queried on 20^th^ of July 2017.

### Topic modelling

Probabilistic topic models were applied to the ~37 k unique publications from the PubMed search query. Stopwords were removed from the text using a standard stopword list and documents with empty abstracts were removed, resulting in a set of ~26k documents and ~4.2 million word tokens, and a vocabulary of ~44 k terms. The Latent Dirichlet allocation collocations model (LDA-COL^28^) was applied to the resulting data set using T = 50, 100, and 200 topics to achieve different levels of granularity in the topics. For each dimensionality, the model was run with standard hyperparameter settings^28^, for 5000 iterations. In contrast to the standard LDA model, the LDA-COL model groups together frequently co-occurring terms into single vocabulary items (e.g., “functional magnetic resonance imaging” is turned into a single term) which facilitates interpretation.

### Labeling structures with cognitive topics

Two different ontologies were used to determine whether a structure can be linked to a certain cognitive topic. The first ontology was based on the classification used in the MeSH hierarchical tree. According to the MeSH description, all terms nested in the node *Psychological phenomena and processes* (MeSH ID: D011579; MeSH tree number: F02) encompass all subtopics that relate to the mechanisms and underlying psychological principles of the mental processes and their applications. All other nodes we classified as non-cognitive topics.

The second ontology that was used to determine whether a structure was related to a cognitive function derived from the probabilistic topic modelling approach. Two independent raters (MCK, MS) manually labelled all topics (across the model dimensionalities of 50, 100, and 200 topics) that related to a cognitive topic. The intraclass correlation coefficient (ICC) in labelling a cognitive topic was 0.71 for the topic model with 50 topics, 0.70 for the topic model with 100 topics, and 0.85 for the topic model with 200 topics, indicating good to excellent agreement between the two raters^29^. The topics that were used in subsequent analyses were those topics judged by both raters as being cognitive, separately for all model runs (with 50, 100, or 200 topics). The 50, 100, and 200 topic models contained 7, 17, and 25 cognitive topics, respectively, for a total of 49 cognitive topics. For each document, we created a vector of 49 topic probabilities that concatenated these probabilities across topic models. Next, for each subcortical structure, we averaged the vector values across all documents that were associated with the subcortical structure. The similarity between subcortical structures was computed on the basis of the correlation between the topic vectors associated the subcortical structures.

### Functional similarity

The MeSH and topic modelling similarity were based on correlations for the 103 structures that had at least one *Psychological phenomena and processes* MeSH term and a cognitive topic model association. Structures that were not associated with any *Psychological phenomena and processes* MeSH term were not considered in these analyses. For each given brain structure, a vector of counts was created representing the number of documents with a particular MeSH term. For each document, the MeSH terms are rolled up through the MeSH hierarchy, meaning that terms lower in the MeSH hierarchy are added to the higher level parent MeSH term count. For any two brain structures (X, Y), the similarity was based on ρ(X, Y), where ρ indicates the correlation between the corresponding vector for structure X and structure Y. This was done for the MeSH vector and psychological topic probability vector separately. To have similar scaling for the MeSH count and psychological topic probability vectors the two similarities were normalized between 0.0 and 1.0.

### Anatomical similarity

**Figure 6:**
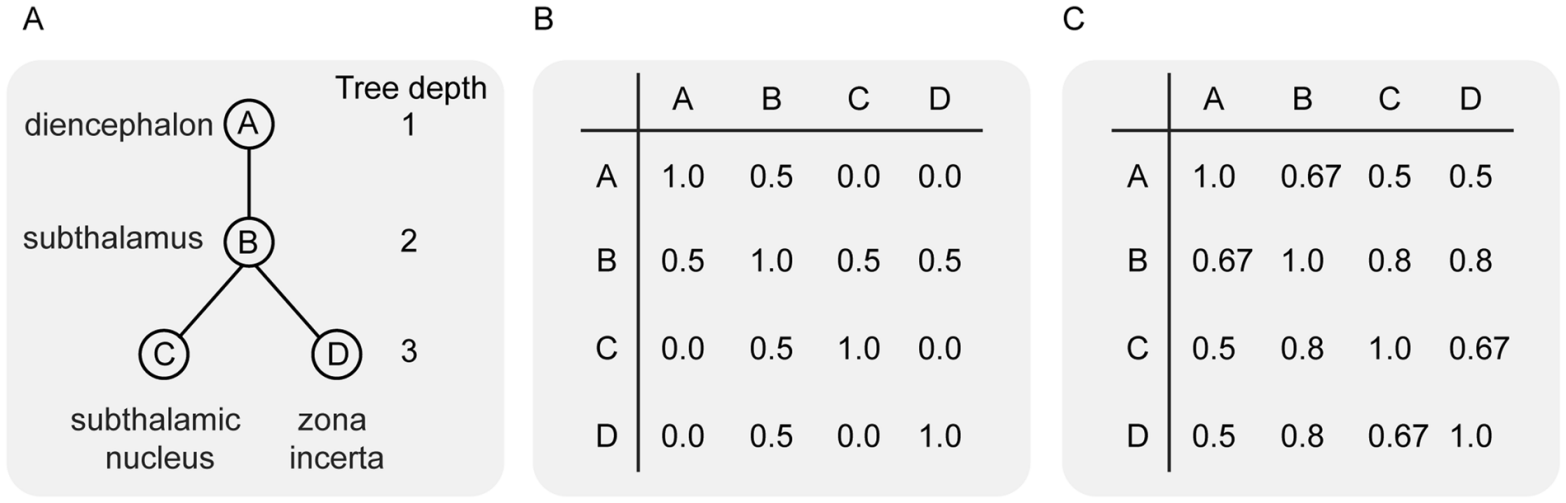
Anatomical similarity examples. (**A**) The hierarchical tree for four subcortical structures. (**B**) The edge counting distance normalized between 0.0 and 1.0 and inverted. (**C**) The inverted anatomical distance normalized by concept hierarchy as proposed by ref.^31^.

The hierarchical anatomical terminology provided by FCAT is organized according to embryonic vertebrate brain development and topographical rules^15^. The anatomical similarity was based on the minimum number of edges in the hierarchical tree between each of the 103 structures that had a *psychological phenomena and process* MeSH term^30^. For instance, for the four structures (a) diencephalon, (b) subthalamus, (c) subthalamic nucleus, and (d) zona incerta, the corresponding hierarchal tree is equal to Fig. 6A.

This is because the subthalamic nucleus and the zona incerta are both child nodes of the subthalamus, which in turn is a child node of the diencephalon. The edge matrix was normalized between 0.0 and 1.0, and inverted so that 1.0 indicates the highest similarity (or shortest distance in the hierarchical tree) and corresponds to Fig. 6B.

As edge-counting does not take the taxonomic level into account, structures that are high in the taxonomic tree can have a similar edge distance as two lower nodes. Therefore, in addition to defining anatomical similarity by edge-counting, we also calculated the similarity by taking the taxonomic hierarchy into account^31^:

$$d(x,y)=\frac{Pmin(x,y)}{l(x)+l(y)}$$

where Pmin(x, y) denotes the edge distance between two structures, and l(x) and l(y) denote the tree depth of the structures in the FCAT taxonomic hierarchy. The concept hierarchy matrix was inverted so that 1.0 indicates the highest similarity and corresponds to Fig. 6C.

### Statistical analysis

The topic modelling was done in Matlab whereas the statistical analyses were done in R^32^. Assumptions of normality were tested using quantile-quantile plots and non-parametric Spearman correlations were used. Due to the large number of data points in the matrices, a significant p-value is not very informative^33^. To test whether the correlations were likely to have occurred due to noise, a sampling procedure was used. We sampled 10,000 times from the anatomical matrix using random permutations and correlated every single matrix with the MeSH or topic model similarity matrix. The sampling of the anatomical distance matrix was done using the R base function *sample*() and resulted in a matrix with identical mean and standard deviations as the original matrix but without the underlying anatomical hierarchy. Afterwards we calculated how many standard deviations (SD) the true correlation was removed from the permutated null distribution.

## Data Availability

All abstracts resulting from the search query, code used to analyze the data, and generate the figures are freely available on osf.io/nf482.
